# Chronic rhinosinusitis in cystic fibrosis: a review of therapeutic options

**DOI:** 10.1007/s00405-021-06875-6

**Published:** 2021-07-22

**Authors:** Joanna Krajewska, Krzysztof Zub, Adam Słowikowski, Tomasz Zatoński

**Affiliations:** 1 Department of Otolaryngology Head and Neck Surgery, Wroclaw Medical University, Wrocław, Poland; 2 Department of Pediatric Pulmonology, Medical Center Karpacz, Karpacz, Poland

**Keywords:** Cystic fibrosis, Otorhinolaryngological manifestation, Rhinology, Sinusitis

## Abstract

**Purpose:**

Chronic rhinosinusitis (CRS) is observed in almost 100% of patients with cystic fibrosis (CF). CF-related CRS treatment is extremely challenging because of the underlying genetic defect leading to its development. CRS in CF is often refractory to standard therapy, while recurrences after surgical treatment are inevitable in the majority of patients. This study provides a precise review of the current knowledge regarding possible therapeutic options for CF-related CRS.

**Methods:**

The Medline and Web of Science databases were searched without a time limit using the terms “cystic fibrosis” in conjunction with “otorhinolaryngological manifestation”, “rhinology” and “sinusitis”.

**Results:**

Precise guidelines for CF-induced CRS therapy are lacking due to the lack of large cohort randomized controlled trials. None of the existing therapeutic agents has already been recommended for CRS in CF. Therapy targeting the underlying genetic defect, intranasal dornase alfa administration, and topical delivery of colistin and tobramycin showed promising results in CF-related CRS therapy. Besides the potential effectiveness of nasal steroids, strong recommendations for their usage in CF have not been provided yet. Systemic corticosteroid usage is controversial due to its potential negative influence on pulmonary disease. Ibuprofen revealed some positive effects on CF-related CRS in molecular and small cohort studies. Intranasal irrigation with saline solutions could relieve sinonasal symptoms. Nasal decongestants are not recommended. Endoscopic sinus surgery is the first-line surgical option for refractory CRS. Extensive surgical approaches should be considered as they could improve long-term outcomes in CRS.

**Conclusion:**

Further studies are warranted to establish consensus for CF-related CRS therapy.

## Introduction

Cystic fibrosis (CF) is a life-shortening autosomal recessive disorder [1]. The prevalence of CF varies widely both by geographic region and by race/ethnicity [2]. In Europe, the occurrence reaches 1/4500 in Western Europe and 1/6000 in Northern and Central Europe, ranging from 1/1353 in Ireland to 1/25.000 in Finland. In Australia, Canada and the USA, the incidence is approximately 1/3000, 1/3300, and 1/4000, respectively. 

CF is less frequent in other geographic regions. In South America, the average incidence is 1/8000 to 1/10.000, ranging from 1/6100 in Argentina to 1/15,000 in Costa Rica. In Afrika and Asia, CF incidence is very low. Among Asian countries, the prevalence is established between 1/10.000 and 1/100.000 in the Indian population. The occurrence is higher in the Middle East than in East Asia, ranging from 1/2560 in Jordan to 1/350.000 in Japan. Reports from Afrika are sparse; nevertheless, the prevalence seems to be higher in the South than in the East and West of Africa [2, 3].It is characterized by the mutations in the cystic fibrosis transmembrane conductance regulator (CFTR), a protein that functions as a chloride and bicarbonate channel. The most common CFTR mutation is ΔF508 [1]. Mutations in the CFTR gene lead to poor chloride and bicarbonate transport through epithelial surfaces and constitutes a main pathogenic mechanism of CF. Improper ion transport results in dehydration of airway surface liquid volume subsequently causing compromised mucociliary clearance (MCC) and multi-organ dysfunction, including progressive pulmonary disease, exocrine pancreatic insufficiency, upper respiratory tract (URT) malfunction, digestive and genitourinary system disorders [4].

The most common URT disorder in CF individuals is chronic rhinosinusitis (CRS) which develops secondary to mucus hyper-viscosity and impaired MCC [1]. CRS negatively influences the lower respiratory tract (LRT) via contributing to pulmonary exacerbations and significantly reduces patients’ quality of life (QOL).

Currently, CF therapy is based on slowing down disease progression and preventing disease-induced long-term dysfunctions. Treatment of CF-related CRS is mainly aimed at reducing and alleviating CRS-related symptoms, as causative therapy for this condition has not already been provided. Management of CF-related CRS is extremely challenging, as this disease is often refractory to standard non-CF CRS treatment. Interventions targeting the underlying genetic defect in the CFTR gene could be revolutionary in CF treatment, nevertheless, their usefulness in CF-related CRS remains unknown and requires further studies.

## Aim of the study

The main aim of this study was to provide a brief review of the recommended therapeutic options for CF-related CRS.

## Methods

The Medline and Web of Science databases were searched without time limit but focusing on the newest report, using the terms “cystic fibrosis” in conjunction with “otorhinolaryngological manifestation”, “rhinology” and “sinusitis”. Auto-alerts in Medline were also considered. The reference lists of original papers and review articles were further searched for additional eligible sources. Articles that did not address the topics were excluded, while the full text of the remaining articles was examined and elaborated on. The search included articles without language limitations.

A total of 1288 articles were originally identified using our search criteria. 1238 articles were excluded after abstract or full-text analysis because they did not exactly address the topic. Therefore, a total number of 50 studies were finally chosen to prepare this manuscript. Studies on which this article was prepared were not limited to large cohorts, as a majority of reports were based on small ones. Because of the low prevalence of CF, lack of large clinical trials, sparse randomized controlled trials (RCTs) on CF-related otolaryngologic diseases, and the lack of precise recommendations for CF-related CRS, we did not exclude non-RTC studies.

## Characteristics of chronic rhinosinusitis (CRS) in CF

### Prevalence

The most common otolaryngological manifestation of CF is CRS. Based on the radiological evaluation, the prevalence of CRS in CF patients reaches up to 100%. CRS with or without nasal polyps (NP) is considered a hallmark of CF-related CRS, and in the pediatric population, CRS with NP (CRSwNP) should always raise suspicion of CF [5]. Data on the geographical distribution of CF-related CRS itself, are missing. However, because of the fact that the prevalence of CRS is almost 100% in CF, the occurrence of sinonasal disease in various geographical regions and ethnicities could presumably be analogous to general distribution of CF. Nevertheless, other risk factors of CRS, including comorbidities, inhaled pollutants, allergens, irritants, toxins, and tobacco smoking that differ in various populations, may influence the actual distribution of CF-related CRS [3]. Development of CRS in CF is mainly related to dysfunctional MCC that develops secondary to CFTR-dependent improper trans-epithelial passage of ions [6]. Dysfunctional MCC leads to obstruction of natural sinuses’ ostia and retention of dense mucus in sinuses. Therefore, a hypoxic environment that predisposes to chronic bacterial colonization of the sinuses is formed [6]. Interestingly, CFTR mutation itself is considered a factor predisposing to CF-related CRS [1].

In general, approximately two-thirds of CF patients develop CRSwNP. Young et al. reported NP in 57% of children aged 5–18 years [7]. CRSwNP in children younger than 6 years, especially if coexisting with LRT or pancreatic dysfunctions, strongly suggests underlying CF [7].

Unlike in the general population with NP, in CF patients, the development of NP is mainly related to neutrophilic and T-helper type 1-dominated inflammatory response that significantly affects the therapeutic approach [8].

### Clinical presentation and symptomatology

The actual frequency of CRS in CF is understated, as the majority of patients do not report CRS symptoms, even though they meet EPOS criteria for CRS and present sinonasal inflammatory changes in imaging. Though sinonasal lesions observed in endoscopy or imaging are very common in CF patients they do not correlate with clinical symptoms. The prevalence of sinonasal changes in imaging in CF reached up to 100%, while only approximately 10–15% of adults with CF, and 20% of children, reported CRS symptoms [1, 9].

Such discrepancy between the number of symptomatic patients and the frequency of inflammatory changes in imaging could result from adaptation to CRS symptoms, a higher burden of LRT dysfunction or the fact that some patients are asymptomatic. Nevertheless, the clear explanation for such a correlation remains unknown [9].

CF patients with CRSwNP typically present nasal obstruction, while those with CRS without NP (CRSsNP), especially adults, more commonly complain of headaches [9]. In contrast, children with CF rarely suffer from headaches and facial pain [7].

Generally, the majority of children with CF-related CRS reported nasal obstruction, rhinorrhea with anterior or postnasal drip, anosmia, and mouth breathing [7]. Similarities between CRS, adenoid hypertrophy, and allergic rhinitis symptoms make the proper diagnosis of CF-related CRS challenging [7].

### Genotype–phenotype correlation

The heterogeneity of genetic defects leading to CF development highlights the complex nature of this disease. It was discovered that the clinical manifestation of CF is determined by the type of the mutation in the CFTR gene and that the degree of CFTR dysfunction implies clinical presentation of the disease [10, 11]. Additionally, genotype appears to influence the severity of CF-related sinonasal disease and presumably may also interfere with the degree of sinus cavity development [12]. Nevertheless, the exact patomechanism via which genotype determines the appearance of CF-related sinonasal disease has not been established yet.

While the most common mutation in CFTR gene is F508del, almost 2000 genetic variants have already been reported [13]. The mutations in CFTR gene were divided into six functional classes according to the mechanism via which they interfere with CFTR gene, subsequently determining the cellular phenotype of CFTR defect [13]. Class I mutations lead to improper synthesis of CFTR, class II mutations result in maturation defects, class III, IV, V and VI mutations lead to gating defect, conductance defect, decreased quantity, and reduced stability, respectively. High-risk genotypes refer to two mutations in class I, II or III. They result in minimal function of CRTF gene and manifest as a more severe form of disease. In contrast, class IV, V and VI mutations lead to residual CFTR function and are responsible for less severe phenotypes [13]. Interestingly, besides the fact the residual CFTR function leads to the classic spectrum of CF, the disease is progressing more slowly than in cases with minimal CFTR activity [13]. The heterogeneity of the clinical presentation of the disease is remarkable as it is determined by the variability in the functional deficit of the CFTR protein in each of the six groups [12].

Almost 90% of CF patients express at least one copy of F508del mutation. According to various authors, homozygosity for F508del was associated with more severe sinonasal disease, the presence of NPs and the higher propensity of endoscopic sinus surgery [10, 11, 14]. Berhout et al. observed that CF individuals with class I–III mutations had significantly less developed frontal and sphenoid sinuses, higher opacification in the sinonasal area, and more frequent presence of bony sclerosis/osteoneogenesis, than those with other class mutations. Lund-Mackay scores were also significantly higher in the group with class I–III mutations [14]. Similar differences in Lund-Mackay scores between patients with high- and low-risk mutations were observed in a study conducted by Ferril et al. [15]. In consistence with these observations, Halderman et al. reported that individuals with high-risk genotypes appeared to have more advanced sinonasal disease, as expressed by higher Lund-Mackay scores, than those with less severe genotypes. Frontal, sphenoid, and maxillary sinus hypoplasia/aplasia was also more commonly observed in patients with high-risk genotypes [12].

Heterozygotes for CFTR gene do not develop typical CF; however; the mutation in one allele could result in the development of CF-like disease [10]. It was reported that carriers of the one mutation in CFRT gene, non-presenting the typical spectrum of CF, were up to five times more prone to develop CRS than non-CF population [10]. Calton et al. observed that CFTR heterozygotes with CRS had significantly smaller volume of the frontal and maxillary sinuses than those without mutations [16]. It was also implied that patients with CRS and a history of juvenile NPs, purulent chronic pansinusitis, frontal and sphenoid sinus hypoplasia, chronic pulmonary dysfunction, or fertility disorders should be examined whether they are CFTR heterozygotes [10].

The difficulty of determining a clear genotype–phenotype correlation regarding CF-related CRS emerges also from the presence of environmental factors and alternative genetic mutations that affect the presentation of sinonasal disease. Mutations indistinct from CFTR genes could modify the clinical presentation of CF-related CRS. For example, homozygotes for F508del and the T2R38 bitter taste receptor genotype expressed better SNOT-22 scores than homozygotes lacking this genotype [10, 11].

Studies analyzing the genotype–phenotype correlation regarding sinonasal disease in CF patients could result in incorporating patient-adjusted therapy aimed at modifying the existing causative genetic defect.

### Imaging

Sinuses CT scan is a useful tool in establishing the extent of CRS and other sinonasal abnormalities. Studies showed that pansinusitis, rather than inflammation limited to particular sinuses, was typically observed in CF patients. The analysis of numerous sinus CT scans in CF patients revealed that hypoplastic sinuses and variants of sinuses lacking pneumatization were significantly more common in CF than in non-CF patients [1].

Besides sinonasal inflammatory changes, imagining may also reveal the presence of “pseudomucocele” in the sinus cavities of CF patients, especially pediatric ones [1]. “Pseudomucoceles” are viscous secretions located in sinuses that do not have true epithelial walls but are limited by a capsule of inflammatory tissues. They develop secondary to osteitis and destruction of the lateral nasal wall or due to pressure exerted on the medial sinus wall by the clusters of thick mucus or polyps [1]. “Pseudomucoceles” occur almost exclusively in the maxillary sinuses in younger children and strongly suggest the presence of CF. In older individuals with CF, instead of “pseudomucoceles”, NPs appear [17].

Sinus hypoplasia is another frequently observed abnormality in both children and adults with CF patients. In CF, hypoplasia typically applies to sphenoid and frontal sinuses. Maxillary sinus hypoplasia could also occur in F508del homozygotes with CF; nevertheless, unlike frontal and sphenoid sinus hypoplasia, it is not characteristic for CF [18].

According to various authors, frontal sinus hypoplasia mainly affected F508del mutation homozygotes, while sphenoid sinus hypoplasia constituted a single criterion that most accurately predicted the presence of CF [18, 19]. Hypo-plastic sinuses mainly result from growth disturbances induced by serious chronic infections or improper embryogenesis related to a genetic mutation. Detection of underdeveloped sphenoid and frontal sinuses in children should always arouse suspicion of CF [1].

It was also reported that the presence of Onodi cells was more common in CF than in non-CF individuals, while Haller cells and concha bullosa were detected less frequently in CF patients. Analyzing the presence of the aforementioned variants is mainly important during the planning and performing sinus surgery in these individuals [1, 18].

### Microbiology and influence on lower airways

It was implied that sinuses constitute a reservoir of pathogens that predisposes to recurrent lung infections and exacerbation of chronic pulmonary disease [1, 5]. The main linking factor between CRS and pulmonary exacerbations is a postnasal drip that carries pathogens to LRT [1]. This association was supported by the results of the microbiological evaluation of the secretions obtained from sinuses and LRT that revealed a similar profile of pathogens in these areas (approximately 80% of homology). *Pseudomonas aeruginosa* and *Staphylococcus aureus* are considered the main pathogens colonizing sinuses in CF patients, while *P. aeruginosa*, via its ability to produce biofilm, is a leading pathogen contributing to persistent airway infection [1]. Additionally, the occlusion of natural sinuses’ ostia by viscous mucus predisposes to chronic bacterial colonization of the sinuses that, in combination with inhaling cold, non-filtered, and dry air through the mouth by patients with CRS and nasal obstruction significantly increase the risk of recurrent exacerbations of pulmonary disease [1, 5].

## CRS-related quality of life in cystic fibrosis

CRS negatively influences QOL in adults with or without coexisting CF. The negative impact of sinonasal dysfunction on patient-reported outcomes (PROs) in CF patients could be even more significant than in non-CF individuals, due to the burden of the systemic nature of CF [5, 20]. The Cystic Fibrosis Questionnaire‐Revised (CFQ‐R) is the most common tool to assess QOL in CF, while up to now, a validated instrument to analyze specifically the influence of sinonasal dysfunction on QOL in children and adults with CF has not been provided [20].

Currently, Sino-Nasal Outcome Test 22 (SNOT-22) is the most widely used questionnaire in CF individuals to evaluate PROs related specifically to rhinologic dysfunctions [20]. Nevertheless, the validation of SNOT-22 in CF has not been provided. However, SNOT-22 appeared to be the most valid and reliable test for the assessment of sinonasal PROs in CF adults, while its utility in children remains unclear [21]. In children aged 2–12 years, The Sinus and Nasal Quality of Life Survey (SN-5) were considered useful in evaluating rhinology-related PROs. However, the validation of the questionnaire covered only non-CF children [21]. Thamboo et al. evaluated the potential usefulness of SNOT-22 in predicting the presence of NP in CF children aged 6–18 years. The authors observed that more than 11 scores in SNOT-22 significantly correlated with the presence of NP suggesting that SNOT-22 could be a useful tool in predicting the presence of NP in children with CF [21].

The positive influence of sinus surgery on QOL in both children and adults with CF was reported by various authors [22].

## Therapeutic options for CF-related CRS

High-level recommendations for CF-related CRS management have not been provided yet, as RCTs investigating various treatment options specifically in the CF population are lacking. Existing studies focusing on CF-related CRS therapy were mainly based on small cohorts. Moreover, the majority of studies did not differentiate between children and adults making the decision of proper age-adjusted therapy even more challenging.

### Conservative treatment (Table 1)

**Table 1:** Juxtaposition of studies analyzing effects of various conservative interventions for CF-related CRS

Type of drug	Type of study	Drug administration	Treatment schedule	Number of patients (*n*)	Age (years)	Outomes	Level of evidence	EBM recommendation in CF-related CRS
Dornase afla [38]	DBPC	Sinonasal inhalation using pulses generated by PARI SINUS	Once a day for 28 days	*n* = 23	5 years and older	In contrast to conventional inhalation significant reduction of rhinosinusitis symptoms was observed Improvement in SNOT-20 scores	I	Not provided
Dornase alfa [40]	DBPC	Intranasal delivery via sidestream nebulizer	Once a day 2.5 mg of dornase alfa for 12 months starting 1 month after sinus surgery	*n* = 12 Patients after FESS	10.6 ± 2.7	Significant increase in FEV1 A persistent improvement in the sinonasal symptoms over the 48 weeks after surgery Improvement in Lund-Mackay scores	I	Not provided
Dornase alfa [41]	Retrospective cohort study	Sinonasal inhalation	Treatment schedule—not reported	*n* = 20 Patients undergoing FESS (5 received dornase alfa)	11–29	Reduction of mucosal edema and no recurrence of NP during over 3 years follow-up The need for revision sinonasal surgery was also decreased during this period No change in pulmonary function tests	III	Not provided
Dornase alfa [42]	Prospective cohort study	Inhaled dornase alfa using PARI SINUS	2.5 mg two times a day for a year	*n* = 103	1–17	Significant shrinkage of NP and improvement in nasal breathing	II	Not provided
Ivacaftor [47]	Case report	Oral	One-month therapy based on daily 150 mg of ivacaftor	*n* = 1 Caucasian woman with long-standing CF-related CRS refractory to conservative and surgical treatment Carrying Mild mutation (P205S) and severe mutation (G551D)	23	Significant clinical improvement in CRS symptoms Reversed changes in sinus CT (almost total resolution of sinus changes of completely obstructed sinuses) Improvement in FEV1 Additional information provided: ivacaftor could reverse the pathogenesis of CF-related sinusitis via reducing airway surface liquid stickiness and enhancing its pH	IV	Not provided
Ivacaftor [48]	Case report	Oral	Dose of ivacaftor and duration of therapy—not reported	*n* = 1 Girl with CF-related CRS refractory to standard treatment with genotype F508del/S1215N	17	Significant reduction of sinonasal symptoms and total resolution of sinuses’ opacification in CT scan	IV	Not provided
Ivacaftor [49]	Case report	Oral	18 months, dose not reported	*n* = 1 Woman with F508del/G551D mutation	19	Complete resolution of CRS symptoms and significant reduction of paranasal sinuses’ changes No pulmonary exacerbations during 1.5 years follow up	IV	Not provided
Ivacaftor [50]	Case series	Oral	6 months, dose not reported	*n* = 12 Patients with G551D-CFTR mutation	10–44	Significant improvement in paranasal sinuses’ pneumatization in CT Improvement in FEV1	IV	Not provided
Ivacaftor [46]	Prospective cohort study	Oral	6 months, dose not reported	129	6 years and older	Significant improvement of the QOL in rhinologic, psychological and sleep domains in SNOT-20 questionnaire at the 1-, 3-, and 6-month follow up	II	Not provided
Ivacaftor [51]	Prospective observational study	Oral	2 months, 150 mg two times a day	*n* = 8 Patients with an S1251N class III gating mutation	9–26	Significant improvement in CF-related sinonasal disease Reduced sinus opacification in CT scans Decrease in pathologic changes in nasal endoscopy Improved sinonasal symptoms and increase of nasal nitric oxide level	II	Not provided
Elexacaftor-tezacaftor-ivacaftor [62]	Retrospective cohort study	Oral	5 months, dose not reported	*n* = *25* Patients with at least one copy of F508del	29.92 ± 12.46	Significant improvement in sinonasal symptoms measured by SNOT-22	III	Not provided
Elexacaftor-tezacaftor-ivacaftor [63]	Retrospective cohort study	Oral	3 months, dose not reported	*n* = 23 Patients with at least one copy of F508del	34 ± 1.8	Significant improvement in sinonasal and respiratory symptoms measured by SNOT-22 and CFQ‐R, respectively Patients undergoing CFTR modulator therapy before triple therapy incorporation had greater improvement than those in whom triple therapy was the initial one	III	Not provided
Ibuprofen [43]	Retrospective cohort study	Oral	40.9 mg/kg/day in 2 doses per day, mean duration 52 months	*n* = 19 Patients with sinonasal disease (12 with NP)	5–15	Resolution of NPs was observed during therapy, nevertheless, ibuprofen-induced effects were only temporary, as, in the majority of patients, NPs reappeared shortly after treatment discontinuation In individuals without NPs prior and during ibuprofen therapy, polyps occurred after cessation of treatment implying the potential protective role of ibuprofen against polyps’ development Authors concluded that long-term, high-dose and weight-adjusted ibuprofen use could be a potential therapeutic option for NPs in CF children	III	Not provided
Tobramycin [30]	Prospective observational study	Nasal irrigation	20 mg of tobramycin added to the last 50 mL of nasal saline irrigation once a day after sinonasal surgery (initiated 10 days after the surgery)	*n* = 37	4–39	Decreased rate of recurrent exacerbations	II	Not provided
Tobramycin [31]	DBPC	Sinonasal delivery via PARI SINUS	tobramycin (80 mg/2 mL) vs. 2 mL isotonic saline once a day for over 28 days	*n* = 9 (6 patients received tobramycin, 3 received placebo)	10.6–38.7	Significant reduction of *P. aeruginosa* colonies in group receiving tobramycin Significant decrease in SNOT-20 scores in group receiving tobramycin	I	Not provided
Combination of various agents [33]	Prospective cohort study	iv. antibiotics in combination with: -nasal irrigation with antibiotic (colistimethate sodium) -nasal irrigation with saline -topical mometasone furoate	Postoperative therapy based on combination of (1) two weeks of broad-spectrum intravenous antibiotics (2) at least 6 months of daily lavage with colistimethate sodium (if susceptible bacteria were cultured) and nasal saline (3) at least 6 months of topical nasal mometasone furoate	*n* = 58 Patients after extensive FESS	6–45	Follow up for 3 years No pathogen growth in sinonasal material during at least 6 months’ follow-up in more than 50% of patients	II	Not provided
Betamethasone [27]	RDBC	Intranasal drops	50 μg of betamethasone in nasal drops twice a day for 6 weeks	*n* = 46	> 16	Significant reduction of the polyps’ dimension Significant reduction of overall sinonasal symptoms	I	Not provided
Sinonasal inhalation NaCl 6.0% or NaCl 0.9% [25]	Multicenter DBPC	Vibrating sinonasal inhalation of either NaCl 6.0% or NaCl 0.9%	Sinonasal inhalation with 4 mL of hypertonic saline (6.0% NaCl) vs. inhalation with isotonic saline once a day for 28 days	*n* = 69	10.8–34.8	No significant difference between sinonasal inhalation with 4 mL of hypertonic saline (6.0% NaCl) and inhalation with isotonic saline once a day for 28 days in CF patients Both therapies resulted in similar improvement in SNOT-20	I	Not provided

DBPC: double-blind placebo-controlled trial; FEV1: forced expiratory volume in 1 s; FESS: functional endoscopic sinus surgery; SNOT-20: Sinonasal Outcome Test-20; NP: nasal polyps; QOL: quality of life; CT: computed tomography; CRS: chronic rhinosinusitis

Conservative therapy is considered the first-line therapeutic option for CF-related CRS and should be incorporated as a primary therapeutic intervention [5]. Studies investigating therapeutic approaches for CF-related CRS are sparse, thus very little evidence concerning the effectiveness of various medications and their recommended dosages currently exists. Even less is known about the effectiveness and safety of CF-related CRS therapy in the pediatric population [5].

According to existing reports, medications that were used in CF-related CRS management included nasal saline irrigations, intranasal and oral corticosteroids, topical and systemic antibiotics, and decongestants. Unfortunately, the usefulness of none of these medications was supported by high-level recommendations [1, 5, 23].

#### Nasal saline lavage

According to the opinion of a multi-disciplinary group of pediatric, otolaryngology, and pulmonology providers, sinonasal irrigation with saline should be recommended in CF-related CRS management [24]. Especially irrigations with isotonic saline (0.9% saline solution) were suggested for sinonasal washing in CF patients [1]. Less is known about the usefulness of sinonasal lavage with hypertonic saline (3% to 7% saline solutions) in these individuals. In non-CF CRS, QOL improved after hypertonic saline irrigations, however, studies focusing specifically on CF patients are lacking [1].

Nasal lavage improves the removal of dried mucus crusts and viscous sinonasal secretions commonly colonized by bacteria. It also facilitates the local penetration of topical medications administered to the sinuses [1].

In contrast to isotonic saline, hypertonic solutions express mucolytic activity on sinonasal mucosa. Osmotic decongestion of mucosa induced by hypertonic saline leads to its shrinkage and could reduce sinonasal obstruction [1, 23]. Due to the retention of high amounts of viscous mucus in sinonasal cavities in CF patients, the saline-induced mucolytic activity could be beneficial in these individuals. Nevertheless, hypertonic solutions should be used with caution as they induce mild and reversible ciliostasis, and irritation of the mucosa [1, 25]. A multi-center RTC conducted by Mainz et al. did not reveal any significant difference between sinonasal inhalation with 4 mL of hypertonic saline (6.0% NaCl) and inhalation with isotonic saline once a day for 28 days in CF patients. Both therapies resulted in similar improvements in SNOT-20 [25]. Currently, the most commonly used solution for sinonasal lavage in CF patients is an isotonic one [1].

#### Corticosteroids

Corticosteroids are immunosuppressive agents whose anti-inflammatory and anti-edematous activity is highly desired in CRS therapy. Their effectiveness in CRS treatment in non-CF patients was well documented [26]. Despite the fact that in the majority of CF patients, dominant inflammatory cells contributing to CRS with NP, are neutrophils, not eosinophils, studies showed that intranasal steroids could also be useful in these individuals [5]. According to the opinion of a multi-disciplinary group of pediatric, otolaryngology, and pulmonology providers, intranasal steroids should be included in CF-related CRS management [24]. However, RCTs analyzing the role of corticosteroids in CF-related CRS are limited, while such studies conducted specifically on the pediatric population are lacking. Strong recommendations for nasal steroid use in CF patients suffering from CRS with or without NP have not been provided yet.

The potential effectiveness of corticosteroids in CF emerges from their anti-inflammatory activity [1]. Corticosteroids reduce nasal discharge and nasal edema, alleviate the evacuation of paranasal sinuses secretions and induce NP shrinkage [23]. It was observed that corticosteroids were able to reduce CRS symptoms, induce NPs’ shrinkage, and prevent the progression of CRS in CF individuals via suppressing the self-perpetuating inflammation-infection cycle triggered by pathogens persistently colonizing sinuses [6, 23].

Topical intranasal administration of steroids eliminates adverse effects of systemic steroids while inducing local therapeutic concentration of the drug, especially if Mygind’s or upside-down positions are used [6, 23].

Generally, neutrophil-mediated CRS that is typically observed in CF patients is less responsive to corticosteroids than eosinophil-mediated CRS [6]. Nevertheless, despite the lack of strong supportive evidence, corticosteroids are also widely prescribed for CF-related CRS.

RCT investigating effects of 50 μg of betamethasone in nasal drops twice a day for 6 weeks in CF-related CRS revealed a significant reduction of the NPs’ dimension and the overall reduction of sinonasal symptoms [27]. It was also suggested that besides typical intranasal steroid delivery, low-absorption lavage with topical steroids could also be a promising way of local drug administration in CF patients [6]. Nevertheless, further studies are needed to confirm this observation.

In selected cases, administration of systemic steroids could be considered, however, unlike in non-CF CRS, little is known about their usefulness and safety in CF-related CRS therapy [23]. Their use in CF patients remains controversial, as oral corticosteroids could lead to exacerbation of the pulmonary disease. Additionally, unlike topical steroids, the systemic ones interfere with the hypothalamic-pituitary axis and predispose to diabetes mellitus that is originally common in CF individuals due to pancreatic insufficiency and metabolic disorder [1, 23].

While short-term use of oral corticosteroids is beneficial in non-CF individuals with CRSwNP, RCTs investigating their influence in CF patients are lacking [1]. There was only a single report presenting that short-term courses of systemic corticosteroids in combination with antibiotics could potentially be useful as the initial treatment for CF-related CRS [1]. In contrast to that, a long course of oral corticosteroids (prednisolone-equivalent dose of 1 to 2 mg/kg) could delay the progression of the pulmonary disease, reduce hospitalizations due to respiratory exacerbations and improve QOL in CF patients [28].

Strong recommendations for or against short and long-term systemic use of corticosteroids in CF individuals have not been provided yet.

#### Antibiotics

The usefulness of topical antibiotics in CRS therapy was proven in non-CF patients [5]. Because of the important role of chronic sinonasal colonization, mainly by *P. aeruginosa* and *S. aureus*, in CF-related CRS, antibiotic therapy seems to be an essential part of CRS treatment in CF patients [5].

Especially topical antibiotics are useful in CRS management, as the concentration of locally administered drugs is high enough to reach therapeutic activity in sinonasal cavities. Simultaneously, serum concentration remains low enough to reduce the risk of drug-induced systemic adverse effects. The most common topical antibiotics used in CRS therapy in CF patients are colistin and tobramycin [29]. Antibiotics can be added to the saline solution during intranasal irrigation or can be administered via inhaler during sinus inhalation [29]. In the CF population, inhalation of antibiotics is considered more effective than their oral or intravenous administration, as inhalative antibiotics penetrate to the sinonasal mucosa more efficiently than systematically administered antibiotics [29]. Moreover, inhalation of antibiotics is safer because it eliminates side effects induced by their systemic use [29].

Davidson et al. reported that 20 mg of tobramycin added to the last 50 mL of nasal saline irrigation once a day after sinonasal surgery decreased the rate of recurrent exacerbations in children and adults with CF [30]. Prolonged aeration of the sinuses was observed in sinus MRI scans in CF patients after sinonasal irrigation with saline and antibiotics. Additionally, nebulizations with tobramycin and colistin were able to prevent the colonization of paranasal sinuses mucosa by bacteria [23]. Nevertheless, studies showed that the standard ways of nebulization did not reach the expected drug concentration in sinuses, as their natural openings were obstructed secondary to CRS [5]. In contrast to standard nebulization, promising results were found for the sinonasal drug delivery using PARI SINUS™ nebulizer [5]. PARI SINUS™ nebulizer is a novel tool, which generates pulses of aerosol that are subsequently delivered to paranasal sinuses [5]. RCT conducted by Mainz et al. revealed that tobramycin (80 mg/2 mL) delivered by PARI SINUS™ to paranasal sinuses once a day for over 28 days led to a significant reduction of *P. aeruginosa* colonies in the majority of CF patients [31]. A significant decrease in SNOT-20 scores was also observed in the studied cohort [31]. Currently, there is also ongoing multi-center double-blind RCT investigating the influence of nasally nebulized tobramycin on sinonasal symptoms, endoscopic scores, QOL, and pulmonary function in CF-related CRS [32].

The influence of the postoperative administration of systemic antibiotics and nasal irrigation with antibiotics on sinonasal bacterial growth in CF patients with CRS undergoing endoscopic sinus surgery (ESS) was studied by Aanaes et al. [33]. Post-operative therapy based on (1) 2 weeks of broad-spectrum intravenous antibiotics, (2) at least 6 months of daily colistimethate sodium and nasal saline lavage, and (3) at least 6 months of topical nasal mometasone furoate, led to no pathogen growth in sinonasal material during at least 6 months’ period in more than 50% of studied children and adults with CF [33].

There is also currently ongoing RTC in a cohort of CF patients analyzing the effectiveness of nasal administration of aztreonam using the PARI SINUS™ nebulizer in combination with oral aerosolized aztreonam vs. intranasal placebo with oral aerosolized aztreonam [34].

#### Macrolides

Macrolides, the group of antibiotics that exerts both, immunomodulatory and antibacterial activity, were considered potentially useful in CRSwNP therapy [35]. Macrolides also stimulate tissue repair via interfering with neutrophil chemotaxis and gathering, mucus production, and its transport through airways [30]. However, the mechanism via which macrolides modulates the host immune system and could positively influence CF-related CRS is complex [35]. A significant decrease in neutrophil, eosinophil, and macrophage amounts, as well as reduction of various molecules, namely neutrophil elastase, interleukin-8 (IL-8), interleukin-6, and tumor necrosis factor-α, was observed in nasal secretions obtained from CF patients with CRS undergoing clarithromycin and roxithromycin therapy [35].

Chronic respiratory tract inflammation in CF patients appears secondary to the release of cytokines by epithelial and immune cells, and the neutrophil influx into airways [35]. Generally, chronic inflammation in CF individuals is mediated by neutrophils and IL-8, thus targeting these elements could play an important role in therapy [29]. It was observed that clarithromycin administered for 2–3 months in non-CF patients with NP led to a significant reduction of NPs’ size and a decrease in IL-8 level in nasal lavage. Such an effect on NP’s size was not achieved in patients with initially low IL-8 levels [29]. The fact that CF patients express elevated IL-8 levels sheds light on the potential therapeutic effect of macrolides on CF-related CRS.

Studies investigating the potential utility of macrolides in CF therapy focused mainly on their impact on chronic pulmonary disease, in which macrolides administration improved survival rates [36]. In CF patients older than 6 years and colonized with *P. aeruginosa*, long-term therapy based on azithromycin reduced the frequency of pulmonary exacerbation and decreased the overall need for additional antibiotics [36].

Macrolides revealed promising effects in non-CF CRS treatment where they were able to reduce CRS-related symptoms, especially nasal discharge, postnasal drip, and nasal obstruction [37].

RCTs analyzing the clinical utility of macrolides specifically in CF-related CRS management are lacking. However, the analogous pathophysiological mechanism contributing to CRS and chronic pulmonary disease in CF implies the potential benefit of these drugs in CF-related CRS.

#### Dornase alfa

Dornase alfa is a mucolytic drug that is able to cleave extracellular long-chain deoxyribonucleic acid (DNA), a thick substance, which appears in high levels in respiratory tracts secondary to neutrophil degradation in CF patients. The usefulness of dornase alfa in CF-related CRS therapy was proven. A prospective double-blind placebo-controlled (DBPC) cross-over trial conducted in 5 years and older CF individuals investigated the effects of sinonasal inhalation of dornase alfa delivered by vibrating aerosol pulses generated by PARI SINUS™ [38]. The results showed that this form of drug administration was able to significantly reduce symptoms of CRS in the CF population in contrast to conventional inhalation that was not efficient enough to reach sinuses. Drug delivery via PARI SINUS™, unlike isotonic saline inhalation, led to significant improvement in sinonasal symptoms and SNOT-20 scores [38]. Positive outcomes observed in this study confirmed similar results achieved by the same researchers in a DBPC cross-over pilot trial [39].

DBPC trial conducted by Cimmino et al. revealed that children with CF receiving dornase alfa via sidestream nebulizer at a dose of 2.5 mg once a day presented a significant improvement in CRS symptoms and Lund–Mackay scores, over the 48 weeks after sinus surgery [40]. Raynor et al. reported that nasally inhaled dornase alfa by CF individuals undergoing ESS resulted in a reduction of mucosal edema and no recurrence of NP during over 3 years of follow-up. The need for revision sinonasal surgery was also decreased during this period [41]. Significant shrinkage of NPs and improvement in nasal breathing were also observed in children aged 1–17 years undergoing therapy based on inhaled dornase alfa using PARI SINUS™ at a dose of 2.5 mg two times a day for a year [42].

#### Ibuprofen

In contrast to ibuprofen-induced delayed deterioration of lung disease in children with CF, the effect of ibuprofen on CF-related CRS has not been widely studied.

The potential role of ibuprofen in CF-related NPs’ therapy was studied in only one clinical research. This study investigated the influence of ibuprofen at a dose of 40.9 mg/kg/day on NPs administered in CF children for approximately 52 months [43]. The resolution of NPs was observed during therapy, nevertheless, ibuprofen-induced effects were only temporary as in the majority of patients, NPs re-appeared shortly after treatment discontinuation [43]. Interestingly, in individuals without NPs before and during ibuprofen therapy, polyps occurred after cessation of treatment implying the potential protective role of ibuprofen against polyps’ development [43]. Lindstrom et al. concluded that long-term, high-dose and weight-adjusted ibuprofen use could be a potential therapeutic option for NPs in CF children [43].

The observed effects of ibuprofen on CRSwNPs could be related to its ability to suppress cyclooxygenase (COX)-1 and COX-2 enzymes that are both upregulated in NPs in CF patients, and related to ibuprofen-induced suppression of neutrophils activation and migration [44].

It was found that ibuprofen could express therapeutic activity in CF via interfering with microtubules’ formation, function and morphology [45]. Molecular investigation revealed that dysfunctional microtubule dynamics in CF cells affected inflammatory signaling and was the main cause of abnormal intracellular transmission. In primary CF nasal epithelial cells, ibuprofen was able to normalize microtubule functioning [45]. RCTs are needed to confirm the potential influence of ibuprofen on CF-related CRS with NPs.

### CFTR modulators

Therapy targeting the underlying genetic defect has already provided promising results in CF therapy. There are two types of CFTR modulators used in CF, namely the so-called correctors that increase transfer of the CFTR to the membrane of the cell surface, and potentiators that improve function of CFTR and transmembrane ion flow. The effect of CFTR modulators on CF-related sinonasal disease was analyzed for ivacaftor in several low-quality studies, and for combined triple therapy based on elexacaftor, tezacaftor, and ivacaftor in two independent investigations. Randomized controlled trials evaluating the role of CFTR modulators in CF-related CRS therapy are lacking, and thus the direct influence of these agents on CF-related sinonasal disease is yet to be established.

#### Ivacaftor

Ivacaftor is a novel gene-based drug approved for the treatment of CF in 6 years old or older patients with at least one copy of G551D mutation in the CFTR gene. Ivacaftor, the so-called “potentiator”, is a therapeutic agent that expresses the ability to improve the ion-channel function of CFTR via the normal cyclic adenosine monophosphate/protein kinase A (cAMP/PKA) signaling pathway. It was observed that ivacaftor was able to improve ion transport at the cellular level leading to improved pulmonary function, QOL, and decreased exacerbation rate in individuals with G551D mutation [46]. However, due to the fact that G551D mutation is observed only in approximately 4–5% of individuals with CF, the potential promising role of ivacaftor in CF therapy is relatively narrow.

Reports of the effect of ivacaftor on CF-related CRS are limited to case reports and case series. Chang et al. presented a case of an adult woman with CF-related CRS refractory to conservative and surgical treatment, in whom significant clinical improvement was achieved after 1-month therapy based on 150 mg of ivacaftor [47]. Recurrence of symptoms was observed when the patient discontinued therapy for 3 days. After 10 months of ivacaftor therapy, a CT scan of paranasal sinuses in this patient revealed almost total resolution of sinus changes that were completely obstructed before the therapy administration. Chang et al. implied that ivacaftor could reverse the pathogenesis of CF-related sinusitis via reducing airway surface liquid stickiness and enhancing its pH [47].

Vreede et al. observed a significant reduction of sinonasal symptoms and total resolution of sinuses’ opacification in CT scan after 5 months of therapy based on ivacaftor in a 17-year-old girl with CF-related CRS refractory to standard treatment [48]. Hayes et al. reported complete resolution of CRS symptoms and significant reduction of paranasal sinuses’ changes after 18 months of ivacaftor therapy in an adult woman with CFTR-G551D mutation [49]. Radiologic improvement in paranasal sinuses’ pneumatization was also reported by Sheikh et al. in a group of individuals with CF-related CRS carrying CFTR-G551D mutation that underwent 6 months of ivacaftor therapy [50]. Additionally, ivacaftor led to significant improvement of the QOL in rhinology, psychological and sleep domains that were evaluated using SNOT-20 questionnaire in 6 years old and older CF patients with G551D mutation at the 1-, 3-, and 6-month follow-ups [46]. Ivacaftor also led to significant improvement in CF-related sinonasal disease in a small cohort of CF patients with an S1251N mutation. The authors observed reduced sinus opacification in CT scans and decrease in pathologic changes in nasal endoscopy, in addition to improved sinonasal symptoms and increase in nasal nitric oxide level [51].

Currently, ivacaftor is only approved for CF-related lung disease, while the approval for CF-related CRS is lacking.

#### Lumacaftor

Lumacaftor acts as a “corrector”. It partially corrects the CFTR misprocessing and subsequently improves the protein transfer to the cell’s surface. Lumacaftor becomes effective when used in combination with ivacaftor, a “potentiator” which reduces the gating abnormality. Lumacaftor–ivacaftor was approved by the US Food and Drug Administration (FDA) for CF treatment in F508del mutation homozygotes aged 2 years and older. Nevertheless, for F508del homozygotes aged 6 to 11 years, a combination of ivacaftor with another “corrector” – tezacaftor is recommended [52].

In randomized DBPC trial conducted by Wainwright et al. on F508del homozygotes aged 12 years and older, lumacaftor–ivacaftor was able to improve pulmonary function (expressed by FEV1), to reduce the risk of pulmonary exacerbations, and to decrease the number of events requiring hospitalization and intravenous antibiotics therapy [53]. Pulmonary function improvement and the reduction of the rate of pulmonary exacerbations in F508del homozygotes aged 12 years and older undergoing lumacaftor–ivacaftor therapy, were also reported recently in another trial [54]. Lumacaftor-ivacaftor did not express significant activity in heterozygotes for the F508del mutation in another clinical trial [55]. The direct influence of lumacaftor and lumacaftor–ivacaftor therapy on CF-related CRS has not already been reported.

#### Tezacaftor

Tezacaftor is a “corrector” and acts similarly to lumacaftor. It is also used in combination with ivacaftor. Tezacaftor–ivacaftor was approved by FDA for patients aged 6 years and older that carry homozygous F508del mutation or at least 1 other mutation that is sensitive to Tezacaftor–ivacaftor [56].

A randomized DBPC trial conducted on F508del homozygotes revealed that therapy based on tezacaftor plus ivacaftor led to modest but significant improvement in FEV1 and in QoL in Cystic Fibrosis Questionnaire-Revised (CFQ-R). It also reduced the rate of pulmonary exacerbations when compared to placebo [56].Tezacaftor–ivacaftor significantly improved FEV1 and CF-related QoF in individuals heterozygous for F508del with a residual function allele when compared to ivacaftor alone and placebo [57].

#### Elexacaftor–tezacaftor–ivacaftor

Elexacaftor is a new generation “corrector” that is aimed at restoring F508del CFTR protein function when used in combination with tezacaftor and ivacaftor [58].Triple combination therapy based on elexacaftor, tezacaftor and ivacaftor was approved for F508del homozygotes and heterozygotes aged 12 years and older, and for patients with any other CFTR gene mutation that is responsive. Triple therapy was recommended for treatment initiation in individuals with genotypes that are responsive for more than one therapy. It means that triple therapy should be used over double and over monotherapy in all individuals that meet the criteria [58]. According to the fact that F508del mutation constitutes the most frequent CFTR mutation, the eligibility for triple therapy in CF exceeds 90% [58].

Combined therapy based on these three CFTR modulators led to significant improvement in chloride transport in human bronchial epithelial cells that was finally able to reach approximately 50% of normal level in F508del heterozygotes and even more in F508del homozygotes [58].

A randomized DBPC trial involving F508del heterozygotes revealed significant improvement in FEV1 and CF-related QoL, in addition to the reduction in pulmonary exacerbations and pulmonary symptoms, in patients undergoing elexacaftor–tezacaftor–ivacaftor therapy [59]. Favorable effects were observed also in patients with advanced lung disease [59]. Another randomized double-blind, active-controlled trial enrolling F508del homozygotes demonstrated that triple therapy based on elexacaftor, tezacaftor and ivacaftor led to more significant improvement in FEV1 and pulmonary symptoms than double therapy based on tezacaftor plus ivacaftor [60].

An observational study conducted on individuals with at least one F508del mutation and advanced pulmonary disease receiving elexacaftor-tezacaftor-ivacaftor treatment showed significant reduction in the number of individuals requiring chronic oxygen therapy and lung transplantation [61]. Additionally, the improvement in pulmonary function was rapid in this research [61].

Reports demonstrating the influence of triple therapy on sinonasal disease in CF are currently limited to only two studies that were conducted on relatively small cohorts of patients with at least one copy of F508del mutation [62, 63]. One of them demonstrated that elexacaftor–tezacaftor–ivacaftor therapy led to significant improvement in sinonasal and respiratory symptoms measured by SNOT-22 and CFQ-R, respectively. Patients undergoing CFTR modulator therapy before triple therapy incorporation had greater improvement than those in whom triple therapy was the initial one [62]. Significant improvement in sinonasal symptoms measured by SNOT-22 was also observed in the other study [63]. Interestingly, there is currently ongoing clinical trial aimed at determining the effects of triple therapy directly on CF-related sinonasal disease; however, not even preliminary outcomes have been provided yet [64].

Because of the fact that CFTR modulators improve the function of the CFTR protein leading to the increase in chloride transmembrane transport and in the reduction of mucus viscosity, they could potentially also interfere with the patomechanism of CRS and thus decrease CRS symptoms in CF. Nevertheless, the direct effect of CFTR modulators on CFrelated CRS requires further studies and elucidation.

#### Nasal decongestants

Nasal decongestants reduce congestion of nasal vessels subsequently decreasing sinonasal edema and improving nasal patency [5]. The therapeutic activity of these agents is lacking. Decongestants must be used with caution as their continuous use for 7 days or more induce drug dependence and iatrogenic rhinitis [5]. Current recommendations opt against nasal decongestants’ use in CRS therapy [26]. RCTs analyzing the usefulness of decongestants in CF-related CRS have not been provided.

### Surgical treatment (Table 2)

**Table 2:** Juxtaposition of studies analyzing effects of surgical intervention for CF-related CRS

Surgical intervention	Type of study	Numer of patients (*n*)	Age (years)	Outcomes	Level of evidence	EBM recommendation
ESS [67]	Nonrandomized prospective clinical trial	*n* = 26 Patients with refractory to conservative treatment CF-related CRS who underwent ESS	3–33	During 23 months follow up Significant reduction of nasal and facial symptoms of CRS after ESS (measured by Likert scales) The recurrence rate of NP after ESS reached 69.2% Postoperatively no polyp recurrence was seen in 30.8%, while improved polyp score in 53.8% patients	II	Not provided
ESS [7]	Prospective cohort study	*n* = 12 Children undergoing ESS	5–16	Radical ESS was recommended over simple polypectomy in children with CF besides the high recurrence rate after both procedures Radical ESS provided significantly longer disease-free intervals (the median interval between repeated ESS reached 4 years and was significantly longer than after polypectomy) Ethmoidectomy and wide opening of the maxillary and sphenoid sinuses in all children are recommended Frontal sinuses surgery not required, as in the majority of CF children frontal sinuses are underdeveloped Reduction of the middle turbinates was recommended to improve postoperative irrigation of sinonasal cavities	II	Not provided
ESS [70]	Retrospective cohort study	*n* = 49 Patients undergoing ESS	2–39	During 6 years follow-up The prevalence of revision ESS was significantly higher in individuals with advanced inflammatory changes in sinus CT scan, and in those with high grade polyps The revision rate of ESS in patients with expansive polyposis reached 58% Patients with minimal polyps did not need revision surgery Patients without NP did not require sinus surgery	III	Not provided
Extended approach consisted of FESS, medial maxillectomy and Caldwell-Luc procedure [73]	Retrospective cohort study	*n* = 15 All patients had at least 3 endoscopic surgical procedures for recurring sinus disease prior the study	9–19	Extended approach was recommended in pediatric population with refractory CF-related CRS initially managed with multiple failed endoscopic procedures Combined approach significantly reduced hospitalization rate due to pulmonary exacerbations, decreased the need for intravenous antibiotic treatment, and improved pulmonary function testing (FEV1%)	III	Not provided
MEMM [22]	Prospective cohort study	*n* = 22	19–43	MEMM led to significant clinical improvement in CF patients, positively influenced QOL related to sinonasal disease and decreased hospitalization rate due to pulmonary exacerbations during 12 months follow-up No improvement in pulmonary function tests after ESS during 12 months of follow-up MEMM with mucosal stripping of maxillary sinuses, and total ethmoidectomy led to significant reduction of the maxillary sinus volume secondary to osteogenesis and auto-obliteration of the sinuses with cancellous bone → decreased mucus retention → reduction of exacerbations rate and the need for revision sinus surgeries The efficacy of postoperative local management of sinus disease appeared to be better after MEMM than after regular maxillary antrostomy	II	Not provided
FESS [75]	Retrospective cohort study	*n* = 21 Patients undergoing fess	4–19	Significant pulmonary improvement lasting 2 years after FESS in CF children	III	Not provided
Aggressive surgical treatment consisting of frontosphenoethmoidectomy, wide opening of all sinuses, extensive removal of bony overhangs and middle turbinates [77]	Retrospective cohort study	*n* = 77 Lung transplant recipients undergoing sinus surgery	26–29.5	Aggressive surgical treatment consisting of frontosphenoethmoidectomy, wide opening of all sinuses, extensive removal of bony overhangs and middle turbinates, resulted in satisfactory effects in adult lung transplant recipients with CF Intraoperative smoothing of bony overhangs, preferably using diamond drill, and wide opening of all sinuses could prevent mucus retention and enable efficient postoperative sinonasal douching Such approach led to significant postoperative reduction of sinonasal colonization by *P. aeruginosa* and *S. aureus* Important role of postoperative daily sinonasal lavage in preventing allograft rejection induced by sinus re-colonization of the CF-relevant bacteria was emphasized	III	Not provided

ESS: endoscopic sinus surgery, FESS: functional endoscopic sinus surgery, MEMM: modified endoscopic medial maxillectomy, CRS: chronic rhinosinusitis, CF: cystic fibrosis, NP: nasal polyps, FEV1%: forced expiratory volume at 1 s (percent)

Surgical intervention is not the first-line treatment option for CF-related CRS, nevertheless, approximately 20–60% of individuals managed conservatively will eventually require Endoscopic Sinus Surgery (ESS) [8].

Up till now, high-level recommendations for surgical management of CF-related CRS are lacking. Functional ESS (FESS) is the most common surgical option for CRS, especially for CRSwNP [7]. Qualifications for ESS in CF patients should follow recommendations for non-CF CRS and be additionally aimed at decreasing lung colonization by bacteria. In general, FESS should be considered in CF individuals with severe sinonasal symptoms not responding to conservative treatment, in those with significant abnormalities in endoscopy or sinus CT, in those with frequent pulmonary exacerbations, and in lung transplant recipients [1]. Bearing in mind rhinologic symptoms, individuals that could presumably benefit most from FESS are those with NP or other anatomic variants that lead to sinus obstruction [1].

Selecting CF patients for sinus surgery is more difficult and challenging than in the general population, because of the relatively high prevalence of sinonasal changes in CT scans that are not accompanied by significant clinical symptoms. CT scan should not be considered a valid criterion for sinus surgery in CF individuals as not all individuals with CT scan changes have symptoms of CRS. A sinus CT scan should rather be used to analyze the anatomy and extent of sinonasal disease in preoperative planning. Additionally, CT should not be routinely used in postoperative follow-up, because of its low accuracy in monitoring disease progression after the surgery [65].

Making a decision whether to perform a sinus surgery in CF patients, especially pediatric ones, is challenging. First, these patients carry an elevated anesthetic risk of complications during surgery performed under general anesthesia [7]. Second, performing ESS in CF patients could be more difficult, as anatomic variants of sinuses’ development and pneumatization that predispose to intraoperative complications during ESS are more frequently observed in CF patients than in the general population. Finally, sinus surgery does not remove the underlying pathogenic mechanisms predisposing to CRS and does not eradicate the sinonasal mucosa disease. Therefore, recurrences seem to be inevitable in these patients, especially in those with severe mutations in the CFTR gene [7]. Individuals with severe CFTR mutations are definitely more likely to undergo ESS than those with mild CFTR mutations [66].

FESS in CF patients is mainly aimed at reducing CRS-related symptoms, nevertheless, the postoperative positive effects are usually temporary [1]. Significant reduction of nasal and facial symptoms of CRS after ESS (measured by Likert scales) was observed in both, children and young adults with CF-related CRS refractory to conservative treatment [67]. In contrast, FESS alone was not able to significantly improve postoperative pulmonary function test (PFT), while, FESS accompanied by postoperative systemic and topical antibiotic therapy led to a significant reduction of bacterial colonization in LRT samples [1].

Studies showed that performing ESS in CF children was relatively safe, and did not lead to significant long-term negative outcomes [68]. Peteghem et al. concluded that extensive FESS did not negatively influence facial growth in CF children, as 10 years of follow-up did not reveal any significant differences in cephalometric measurements in these patients [68].

In selected, less severe cases of CRSwNP, especially in children, polypectomy could be considered as an alternative to ESS [1, 7]. However, besides being a less invasive procedure than ESS, the recurrence rate after polypectomy exceeded 80% in CF patients, while the postoperative reduction of sinonasal symptoms was short-lasting [69]. RTCs comparing ESS and simple polypectomy in the pediatric population with CF-related CRS are lacking. Therefore, a decision on whether to perform a polypectomy instead of a more radical procedure must be made with caution [1].

Yung et al. recommended radical ESS over simple polypectomy in children with CF besides the high recurrence rate after both procedures [7]. Nevertheless, radical ESS provided significantly longer disease-free intervals. The median interval between repeated ESS reached 4 years and was significantly longer than observed after polypectomy. The authors recommended ethmoidectomy and a wide opening of the maxillary and sphenoid sinuses in all children. Frontal sinuses surgery is usually not required, as in the majority of CF children frontal sinuses are underdeveloped. Simultaneous reduction of the middle turbinates was recommended to improve postoperative irrigation of sinonasal cavities [7].

Interestingly, it was observed that the size of NP at the time of the first ESS constituted a significant predicting factor of the revision surgery in both children and adults with CF [70]. The prevalence of revision ESS was significantly higher in individuals with advanced inflammatory changes in sinus CT scan, and those with expansive polyps. The revision rate in the latter group reached 58%. In contrast, patients with minimal polyps did not need revision surgery within 6 years of follow-up, while individuals without NP did not require sinus surgery during this period at all [70].

The recurrence rate of NP after ESS in children and adults with CF reached 69% during the mean 23 months’ follow-up in another research. Postoperatively, 15% of studied patients developed the same extent of polyposis, while in 53.8% of patients, improved polyp score was reported [67]. No recurrence of sinus mucoceles after ESS during 3 months’ to 6 years’ follow-up was observed in the pediatric population with CF in another study [71].

Properly performed ESS must always be accompanied by adequate postoperative sinonasal irrigation, debridement, and administration of topical medications. Aanaes et al. used single-photon emission CT to assess the postoperative range of saline penetration to the sinuses administered during sinonasal lavage in CF patients. They observed that no saline penetrated sphenoid and frontal sinuses, while maxillary sinuses were reached in less than 50% of cases [72]. This observation could partially explain the relatively rapid recurrence after ESS in some CF patients [72].

## Extended procedures/combined approaches

In patients with a history of recurrent ESSs, extended surgical procedures or combined surgical approaches should be considered. Studies showed that aggressive surgical treatment was beneficial in CF individuals, as it reduced the need for revision surgeries, provided better administration of topical medications, and better control of chronic bacterial colonization of the sinuses. It was reported that this approach could be especially beneficial in CF patients with high Lund–Mackay scores at their initial CT scan, as these individuals were more likely to require revision sinus surgeries [8, 22, 73].

Among all paranasal sinuses, aggressive surgical treatment performed on maxillary sinuses is a priority in CF patients, as maxillary sinuses, due to their superiorly localized ostia, constitute the main reservoir of bacteria in URT [8]. Effective mucus evacuation from maxillary sinuses after properly performed maxillary antrostomy might not be achieved in CF individuals because of the rapid postoperative re-accumulation of viscous mucus, even despite nasal irrigations [8].

Shatz et al. recommended a combined approach consisting of FESS, medial maxillectomy and Caldwell-Luc procedure in the pediatric population with refractory CF-related CRS initially managed with multiple failed endoscopic procedures [73]. Such an approach significantly reduced hospitalization rate due to pulmonary exacerbations, decreased the need for intravenous antibiotic treatment, and improved pulmonary function testing (defined as forced expiratory volume at 1-s percent [FEV1%]) [73].

Modified endoscopic medial maxillectomy (MEMM), a procedure currently recommended for recalcitrant maxillary sinus disease, led to significant clinical improvement in CF patients, positively influenced QOL related to sinonasal disease, and decreased hospitalization rate due to pulmonary exacerbations during 1-year follow-up. Additionally, MEMM with mucosal stripping of maxillary sinuses, and total ethmoidectomy performed in CF children led to a significant reduction of the maxillary sinus volume secondary to osteogenesis and auto-obliteration of the sinuses with cancellous bone. Such structural remodeling significantly decreased mucus retention, and could subsequently reduce the risk of exacerbations and the need for revision sinus surgeries [22].

The efficacy of postoperative local management of sinus disease appeared to be better after MEMM than after regular maxillary antrostomy, while the risk of revision operation seemed to be reduced [22].

It is known that sinonasal mucosa in CF would never function properly because of the underlying genetic defect. Therefore, aggressive surgical treatment aimed at creating wide and permanent “access” to the sinuses enabling easier and more effective postoperative irrigation, debridement and drug deposition, could improve long-term outcomes in CF-related CRS [8, 22, 73].

## Influence of sinus surgery on LRT

Besides focusing on reducing sinonasal symptoms, sinus surgery in CF patients is also aimed at improving pulmonary function [74]. The outcomes of sinus surgery in CF adults are mainly evaluated using the FEV1 parameter that is considered the main marker reflecting successful intervention [74].

Kovell et al. reported significant pulmonary improvement lasting 2 years after FESS in CF children [75]. No betterment in pulmonary function tests after ESS during 12 months of follow-up was also observed by other authors, however, the hospitalization rate due to pulmonary exacerbations was significantly reduced during 1-year follow-up [22].

FEV1 usefulness in the pediatric population is not clear, as its decrease, reflecting improvement, is usually observed after reaching adolescence [76]. It could presumably influence the results of FEV1 analyses after sinus surgery in these patients.

## The usefulness of CT in postoperative monitoring

Sinus CT scan should not be considered useful in predicting outcomes after surgical management in CF patients, as Lund–MacKay scores before and after surgery did not significantly differ in these individuals, in contrast to the reduction of symptoms and improvement in QOL. Repetitive postoperative sinus CT scans are not recommended in monitoring CF-related CRS progression, as they do not correspond with patients’ symptoms and clinical exacerbations [65].

## Sinus surgery in lung transplant recipients with CF

It is known that a number of CF patients will develop end-stage pulmonary disease and will finally require lung transplantation. *P. aeruginosa*, chronically colonizing sinuses in CF patients, predisposed to bronchiolitis obliterans syndrome, played a critical role in the progression of CF-related pulmonary disease, and could negatively influence the survival rate after lung transplantation [44]. In lung transplant recipients, chronic pulmonary colonization by *P. aeruginosa* is usually eradicated, however, the pathogen could still colonize sinuses and, when transmitted to LRT, could induce infection and rejection of the graft [44].

It was implied that successful sinus operation could reduce the risk of these post-transplant complications induced by bacteria colonizing paranasal sinuses [1]. Nevertheless, the clear association is yet to be elucidated [77].

It was reported that the re-hospitalization rate due to pulmonary dysfunction was significantly reduced in lung transplant recipients undergoing FESS. Post-transplant sinus surgery accompanied by daily sinonasal lavage decreased the prevalence of tracheobronchitis, pneumonia and bronchiolitis obliterans syndrome [44]. Vital et al. reported that aggressive surgical treatment consisting of frontosphenoethmoidectomy, wide opening of all sinuses, extensive removal of bony overhangs and middle turbinates, resulted in satisfactory effects in adult lung transplant recipients with CF. According to Vital et al., intraoperative smoothing of bony overhangs, preferably using the diamond drill and a wide opening of all sinuses could prevent mucus retention and enable efficient postoperative sinonasal douching. Such an approach led to a significant postoperative reduction of sinonasal colonization by *P. aeruginosa* and *S. aureus*. They emphasized the important role of postoperative daily sinonasal lavage in preventing allograft rejection induced by sinus re-colonization of the CF-relevant bacteria [77].

## Conclusion

Precise recommendations for CF-related CRS therapy are lacking. Currently, management of CF-related CRS is mainly based on recommendations provided for non-CF CRS. Generally, CF-related CRS treatment should be mainly conservative. In cases of severe sinonasal symptoms despite conservative treatment or in those with CRS-related complications, surgical treatment is needed. The answer to the question, whether to perform prophylactic sinus surgery after lung transplantation or not remains ambiguous.

Due to the fact that the prevalence of relapses in CF patients undergoing sinus surgery is high because of the underlying genetic defect leading to CRS, a number of patients would subsequently require revision surgeries. It explains why surgical treatment should not be considered a first-line treatment for CF-related CRS, while the decision of whether to perform surgical intervention must be made with caution. FESS is the most common surgical procedure, however, extended surgical approaches seemed to provide better long-term outcomes in CF individuals.

As the life expectancy of CF patients is constantly increasing and CRS is one of the most common CF-related disorders, further multi-center large cohort RCTs with long-lasting follow-up are warranted to establish the best therapeutic options for these individuals.
